# Lipoprotein(a) and incident type-2 diabetes: results from the prospective Bruneck study and a meta-analysis of published literature

**DOI:** 10.1186/s12933-017-0520-z

**Published:** 2017-03-21

**Authors:** Ellie Paige, Katya L. Masconi, Sotirios Tsimikas, Florian Kronenberg, Peter Santer, Siegfried Weger, Johann Willeit, Stefan Kiechl, Peter Willeit

**Affiliations:** 1 Department of Public Health and Primary Care, University of Cambridge, Cambridge, UK; 2 Department of Medicine, University of California San Diego, La Jolla, CA USA; 3 Division of Genetic Epidemiology, Medical University of Innsbruck, Innsbruck, Austria; 4 Department of Laboratory Medicine, Bruneck Hospital, Bruneck, Italy; 5 Department of Internal Medicine, Bruneck Hospital, Bruneck, Italy; 6 Department of Neurology, Medical University of Innsbruck, Innsbruck, Austria

**Keywords:** Lipoprotein(a), Diabetes, Prospective study, Meta-analysis

## Abstract

**Aims:**

We aimed to (1) assess the association between lipoprotein(a) [Lp(a)] concentration and incident type-2 diabetes in the Bruneck study, a prospective population-based study, and (2) combine findings with evidence from published studies in a literature-based meta-analysis.

**Methods:**

We used Cox proportional hazards models to calculate hazard ratios (HR) for incident type-2 diabetes over 20 years of follow-up in 815 participants of the Bruneck study according to their long-term average Lp(a) concentration. For the meta-analysis, we searched Medline, Embase and Web of Science for relevant prospective cohort studies published up to October 2016.

**Results:**

In the Bruneck study, there was a 12% higher risk of type-2 diabetes for a one standard deviation lower concentration of log Lp(a) (HR = 1.12 [95% CI 0.95–1.32]; P = 0.171), after adjustment for age, sex, alcohol consumption, body mass index, smoking status, socioeconomic status, physical activity, systolic blood pressure, HDL cholesterol, log high-sensitivity C-reactive protein and waist–hip ratio. In a meta-analysis involving four prospective cohorts with a total of 74,575 participants and 4514 incident events, the risk of type-2 diabetes was higher in the lowest two quintiles of Lp(a) concentrations (weighted mean Lp(a) = 3.3 and 7.0 mg/dL, respectively) compared to the highest quintile (62.9 mg/dL), with the highest risk of type-2 diabetes seen in quintile 1 (HR = 1.28 [1.14–1.43]; P < 0.001).

**Conclusions:**

The current available evidence from prospective studies suggests that there is an inverse association between Lp(a) concentration and risk of type-2 diabetes, with a higher risk of type-2 diabetes at low Lp(a) concentrations (approximately <7 mg/dL).

**Electronic supplementary material:**

The online version of this article (doi:10.1186/s12933-017-0520-z) contains supplementary material, which is available to authorized users.

## Background

Elevated lipoprotein(a) [Lp(a)] concentrations have been implicated in cardiovascular disorders, with strong evidence from prospective cohort studies and Mendelian randomisation (MR) studies suggesting a causal relationship between high concentrations of Lp(a) and increased risk of cardiovascular disease (CVD) [1–4]. In contrast, its relationship with incident type-2 diabetes is less clear. The first prospective study on this topic [5] showed evidence of an inverse relationship between Lp(a) concentration and incident type-2 diabetes. Participants in the highest quintile (>45.3 mg/dL) of Lp(a) concentration had a lower risk of type-2 diabetes compared to those in the lowest quintile (<3.9 mg/dL) of Lp(a) (HR = 0.78 [95% CI 0.67–0.91]). Since then, three additional prospective studies have reported findings consistent with an inverse relationship between Lp(a) levels and incident type-2 diabetes [6–9]. The question of whether this is a causal relationship is of clinical importance since drugs that selectively reduce Lp(a) are now available [10].

Lp(a) is composed of an LDL-like particle linked to apolipoprotein(a) [apo(a)] [11]. Blood concentrations vary widely among individuals and are highly skewed rightward at the population level [11]. Lp(a) levels are primarily under genetic control, with genetic variants and the number of kringle IV-2 (KIV-2) repeats in the *LPA* gene accounting for much of the variability in Lp(a) concentrations [11]. KIV-2 repeats are translated into apo(a) isoforms with low numbers of repeats resulting in small apo(a) isoforms and higher numbers of repeats resulting in large apo(a) isoforms [11].

Unlike observational studies which can be affected by residual confounding and reverse causality, MR studies use genetic variants as proxies for the exposure to assess causality [12]. Under the principle of independent assortment, genetic variants are not associated with confounding factors and are not subject to reverse causality when assessed in relation to a disease outcome since variants are randomly assorted during meiosis [13]. Two MR studies have assessed the relationship between Lp(a) and type-2 diabetes and found a null association between the variant rs10455872, mainly found in European cohorts, and risk of incident diabetes [6, 9]. The rs10455872 variant is associated with small apo(a) isoforms and high Lp(a) levels [11] and has been previously shown to be strongly associated with an increased risk of CVD [14]. However, rs10455872 tags individuals with a wide range of Lp(a) concentrations and is suggested to be an imprecise proxy for assessing causality between low Lp(a) levels and diabetes [11, 15]. The MR study by Kamstrup and Nordestgaard [6] also assessed the sum of KIV-2 repeats of both apo(a) alleles, finding that the highest quintile of the sum of KIV-2 repeats, which are associated with larger apo(a) isoforms and lower levels of Lp(a), were associated with a 16% increased risk of type-2 diabetes compared to the lowest four quintiles (OR 1.16 [95% CI 1.05–1.28]) [6]. A causal relationship between low levels of Lp(a) concentration and increased risk of type-2 diabetes cannot be ruled out based on these findings.

The aims of the present study were two-fold. First, to examine the relationship between long-term average levels of Lp(a) concentration and incident type-2 diabetes in the Bruneck study. Second, to conduct a meta-analysis combining results from the Bruneck study with results from the previously published studies.

## Methods

### Bruneck study population

The Bruneck Study is a prospective population-based survey of the epidemiology and pathogenesis of atherosclerosis [16, 17]. The study design and survey have been described in detail elsewhere [18–20]. Briefly, in 1990, a random sample of 1000 men and women aged 40–79 years living in the city of Bruneck (125 per sex and decade of age) were invited to participate in the study. A total of 936 men and women were enrolled and re-examined every five years since. The participants in this study had access to one public health care system, the Bruneck Hospital which is the only health care provider in the region. The network existing between hospital and practitioners allowed for the retrieval of full medical information of all individuals. The follow-up rate was 99.9% regarding clinical end points.

### Ascertainment of type-2 diabetes

At baseline, type-2 diabetes was diagnosed using the World Health Organization (WHO) criteria [21]; a fasting glucose ≥126 mg/dL, a 2-h oral glucose tolerance test (OGTT) ≥200 mg/dL after a standard 75-g glucose load or a clinical diagnosis of diabetes with the use of medication and/or diet modification. At subsequent follow-ups, OGTTs were not performed. Instead, diagnosis of type-2 diabetes was ascertained using the American Diabetes Association (ADA) criteria [22].

### Clinical and laboratory data

At baseline and each 5-year follow-up, sociodemographic characteristics, comorbidities and medication use were recorded. Data were retrieved with a clinical history and medical record review, a complete physical examination, and laboratory measures. Body mass index (BMI), waist–hip ratio (WHR), smoking status, alcohol consumption and blood pressure were assessed using validated standard procedures. Physical activity was recorded by composed score for work (three categories) and sports/leisure activities (0, ≤2, and >2 h/week). Socioeconomic status (SES) was categorized on a three-category scale (low, medium, and high) based on information about occupational status and educational level of the person with the highest income in the household. Venous blood samples were drawn after an overnight fast and abstinence from smoking. Total cholesterol, low density lipoprotein cholesterol (LDL-C), high density lipoprotein cholesterol (HDL-C), and high-sensitivity C-reactive protein (hsCRP) were all assessed by standard methods. Since LDL-C was estimated with the Friedwald formula and is therefore affected by the cholesterol content of Lp(a), we calculated corrected LDL-C by subtracting Lp(a) mass in mg/dL and multiplying by 0.45 [23]. Lp(a) was measured in fasted samples by a double-antibody ELISA (Immuno) using a polyclonal anti-apo(a) for capture and a monovalent anti-apo(a) Fab fragment coupled with peroxidase for detection. Lp(a) testing was repeated in 1995 using an updated, more sensitive method. As previously described [24], apo(a) phenotyping was performed by sodium dodecyl sulfate–agarose gel electrophoresis (SDS agarose) under reducing conditions [23].

### Bruneck study statistical analysis

We analysed data from individuals without a baseline diagnosis of diabetes and with Lp(a) concentration measured at baseline. Baseline characteristics of participants were examined. Continuous variables were presented as mean (standard deviation [SD]) or medians (interquartile range [IQR]) and dichotomous variables as percentages. Linear regression was used to determine the percentage mean difference in log transformed baseline Lp(a) levels per SD increase in the baseline characteristics. To account for the skewed distribution of Lp(a) concentration, we log-transformed Lp(a) levels and estimated usual levels by regressing the log-transformed Lp(a) values measured at the 5-year follow-up on the log-transformed Lp(a) baseline values [25, 26]. Participants were grouped into Lp(a) categories based on these usual values and cut-points defined by quintiles of baseline Lp(a) concentration.

Cox proportional hazards regression was used to estimate adjusted hazard ratios (HR) and 95% confidence intervals (CI) for the risk of diabetes per SD lower log Lp(a) level and by quintiles of Lp(a) concentration, with quintile 5 as the reference group. A 1-SD lower log Lp(a) level corresponded to a 3.5-fold difference on the original scale (i.e., e^1.3^). Model 1 was adjusted for age and sex; Model 2 was further adjusted for alcohol consumption, BMI, smoking status, SES and physical activity; and Model 3 was further adjusted for systolic blood pressure, HDL-C, log hsCRP and waist–hip ratio. We also investigated the association between apo(a) isoform size (large isoforms vs. small [reference group] using a cut-point of 22) and incident diabetes risk using Cox models and adjusting for the same covariates used in Model 3.

We investigated the potential role of reverse causality and residual confounding in the Lp(a)-diabetes relationship using a series of sensitivity analyses. We re-ran Model 3 with (1) additional adjustment for fasting glucose; (2) additional adjustment for HbA1c; (3) additional adjustment for fasting glucose and HbA1c; (4) additional adjustment for apo(a) level; (5) additional adjustment for corrected LDL-C; (6) including only Lp(a) measurements assessed at the 5-year follow-up; (7) excluding people with HbA1c >6.5%; and 8) excluding the first 5 years of follow-up.

We further examined the potential influence of reverse causality using Model 3, plotting time-dependent HRs for Lp(a), with 95% CIs. The data were broken into time-dependent strata (2 year intervals) for Lp(a), and the coefficient for each time interval accounted for individuals from that time point until the end of follow-up, while the coefficients of the remaining covariates are assumed to be constant across strata. The proportional-hazards assumption for each covariate in Model 3 with time-dependent intervals for Lp(a) was tested. Statistical analyses were performed in R Statistical Software (version 3.3.1) [27].

### Meta-analysis search strategy and selection criteria

We identified studies published from 1975 to October 2016 through a systematic literature search of Medline, Embase and Web of Science using the terms “diabetes” or “diabetes mellitus” or “Diabetes Mellitus”[Mesh] (MEDLINE only), and “lipoprotein(a)” or “Lp(a)” or “Lipoprotein(a)”[Mesh] (MEDLINE only). We restricted our search to articles published in the English language and reporting on humans.

All prospective cohort studies that: (1) reported on the association of Lp(a) and incident type-2 diabetes; and (2) did not select participants on the basis of having any previous chronic disease were eligible for inclusion in the study. A data extraction form was used to record patient and study characteristics, including sample size, number of diabetes cases, mean or median follow-up time, number and percent of males, mean or median age, Lp(a) ascertainment and diabetes ascertainment. For incident type-2 diabetes outcomes, summary statistics (HRs or odds ratios [OR] and 95% CIs) for each Lp(a) quintile were extracted. When a study had reported effect estimates for different levels of adjustment, we extracted the estimate relating to the most fully adjusted model. Data were extracted by one investigator (EP), independently checked by another investigator (KM) and discrepancies were resolved by discussion and by adjudication of a third investigator (PW).

### Meta-analysis statistical analysis

Effect sizes and standard errors for the risk of diabetes were pooled across the studies using fixed-effect meta-analysis weighted by the inverse variance. Only studies reporting incident diabetes risk according to quintiles of Lp(a) concentration were included in the meta-analysis. Four meta-analyses were run with each quintile of Lp(a) concentration from 1 to 4 treated as a separate outcome and compared to quintile 5. Effect sizes and standard errors were first log-transformed and studies reporting effect sizes in relation to quintile 1 were transformed to give effect sizes in relation to quintile 5. Heterogeneity between studies within each Lp(a) quintile was quantified using the I^2^ statistic [28]. Weighted mean Lp(a) concentrations in each quintile were approximated by taking the average of the median Lp(a) concentrations in each quintile, weighted by the number of participants in each study, for those studies that reported median Lp(a) concentrations by quintile. Since quintiles of Lp(a) concentration are not independent, we undertook a sensitivity analysis using multivariate meta-analysis which can combine effect sizes from multiple studies across related parameters [29–31]. Most published studies do not report covariance matrices needed for estimating within-study correlation in multivariate meta-analysis so we fitted three models in which we changed the within-study correlation to reflect a range of plausible values (0.2, 0.5 and 0.8). Analyses were undertaken using Stata version 14.2.

## Results

### Bruneck study

Baseline characteristics for the 815 Bruneck participants with recorded baseline Lp(a) measurements and without diabetes at baseline are provided in Table 1. Mean age was 58 years (SD = 11) and 403 (50%) participants were men. The median baseline Lp(a) level for individuals was 8.9 mg/dL (IQR 4.5–22.2). In a cross-sectional analysis, Lp(a) concentration was associated with the number of KIV repeats, LDL-C corrected for Lp(a)-cholesterol, and HbA1c (adjusted mean differences in Lp(a): −38.0, +26.0, +12.0%, respectively; all P ≤ 0.01).

**Table 1:** Baseline characteristics of participants and cross-sectional associations with lipoprotein(a) (n = 815)

Baseline variables	Mean (SD), median (25th–75th), or n (%)	Age- and sex-adjusted % mean difference in Lp(a) (95% CI) per SD higher level or compared to reference group
Baseline Lp(a), mg/dL^a^	8.9 (4.5–22.2)	–
Apo(a) isoform size, KIV repeats	25 (4.5)	−38.0 (−41.0, −34.0)**
Age, mean (SD), years	58 (11.0)	1.3 (−4.6, 7.6)
Male sex, %	403 (49.5)	−2.1 (−13.2, 10)
Current smoking, %	199 (24.4)	13.0 (−1.8, 30.0)
Alcohol consumption, gram/week	10 (0–50)	−1.4 (−8.0, 5.7)
Physical activity, Baecke score		
Low	148 (18.2)	[Reference]
Medium	312 (38.3)	−16.0 (−29.0, 0.65)
High	355 (43.6)	0.1 (−16.0, 19.0)
SES		
Low	504 (61.8)	[Reference]
Medium	173 (21.2)	−0.1 (−14.0, 17.0)
High	138 (16.9)	−17.0 (−30.0, −1.0)
Systolic blood pressure, mmHg	144 (21.0)	1.6 (−5.0, 8.6)
BMI, kg/m^2^	25 (3.7)	2.3 (−3.7, 8.7)
WHR	0.89 (0.07)	4.9 (−2.5, 13.0)
LDL-C, mg/dL^b^	137 (38.0)	26.0 (19.0, 33.0)**
Triglycerides, mg/dL	130 (78.4)	1.9 (−7.6, 4.3)
hsCRP, mg/L	0.14 (0.084–0.28)	0.3 (−5.7, 6.6)
HOMA-IR	2.5 (1.6–3.7)	−1.6 (−7.4, 4.7)
HbA1c, %	5.4 (0.4)	12.0 (5.3, 20.0)*
Fasting glucose, mg/dL	97 (9.5)	0.7 (−5.3, 7.1)

*BMI* body mass index, *HDL-c* high density lipoprotein cholesterol, *HOMA-IR* homeostatic model assessment-insulin resistance, *hsCRP* high sensitivity c-reactive protein, *KIV* kringle IV, *SD* standard deviation, *SES* socioeconomic status, *WHR* waist hip ratio

* P ≤ 0.01

** P ≤ 0.001

^a^Single measurement in 1990

^b^LDL-C corrected by removing Lp(a) multiplied by 0.45 [23]

Over 20 years of follow-up, 94 incident events of type-2 diabetes were recorded. The HRs for type-2 diabetes, according to Lp(a) quintiles, are shown in Table 2. After adjusting for age and sex, there was an increased risk of type-2 diabetes for those with Lp(a) concentrations in quintile 2 (median Lp(a) = 5.1 mg/dL) compared to quintile 5 (51.9 mg/dL) (HR = 1.90 [1.04–3.45]; Table 2). Although the HRs were higher for quintiles 1 (2.3 mg/dL), 3 (8.8 mg/dL) and 4 (17.5 mg/dL) compared to quintile 5, these results were not statistically significant. HRs strengthened somewhat following further adjustments in Models 2 and 3, but the overall patterns remained the same. In the most fully adjusted model, Model 3, those in second quintile of Lp(a) concentration had 2.24-times the risk of developing type-2 diabetes than those with the highest Lp(a) levels (HR = 2.24 [1.22–4.10]; Table 2). When Lp(a) was included in the model as a linear predictor, a standard deviation lower log Lp(a) concentration was associated with a 12% higher type-2 diabetes risk (HR = 1.12 [0.95–1.32], P = 0.171; Table 2) in Model 3. In analyses focusing on apo(a) isoform size as an exposure, we found no association with incident type-2 diabetes (HR comparing small vs. large isoforms = 1.01 [0.97–1.06], P = 0.584).

**Table 2:** Hazard ratios (95% CI) for risk of incident type-2 diabetes according to usual levels of lipoprotein(a) concentration

	No. incident type 2 diabetes cases	Median (range), Lp(a), mg/dL	HR (95% CI) Model 1	P value	HR (95% CI) Model 2	P value	HR (95% CI) Model 3	P value
Quintile								
1	20	2.3 (0.8–3.6)	1.36 (0.74, 2.48)	0.326	1.35 (0.73, 2.49)	0.336	1.37 (0.74, 2.53)	0.311
2	26	5.1 (3.7–6.6)	1.90 (1.04, 3.45)	0.036	2.15 (1.18, 3.93)	0.013	2.24 (1.22, 4.10)	0.009
3	17	8.8 (6.7–12.3)	1.42 (0.76, 2.66)	0.269	1.34 (0.71, 2.51)	0.365	1.43 (0.75, 2.71)	0.276
4	18	17.5 (12.5–26.9)	1.05 (0.53, 2.08)	0.896	1.02 (0.51, 2.03)	0.961	1.01 (0.51, 2.01)	0.981
5	13	51.9 (27.1–316.2)	[Reference]		[Reference]		[Reference]	
Per SD lower log Lp(a)			1.10 (0.93, 1.29)	0.253	1.12 (0.95, 1.32)	0.176	1.12 (0.95, 1.32)	0.171

Model 1 was adjusted for age and sex. Model 2 was additionally adjusted for alcohol consumption, BMI, smoking, SES and physical activity. Model 3 was adjusted for the same factors as Models 1 and 2 plus systolic blood pressure, HDL-C, log hsCRP and waist–hip ratio

Usual Lp(a) concentration are predicted long-term average levels of Lp(a) estimated by regressing the log-transformed Lp(a) values measured at the 5-year follow-up on the log-transformed Lp(a) baseline values

*CI* confidence intervals, *BMI* body mass index, *HDL-C* high density lipoprotein cholesterol, *HR* hazard ratio, *hsCRP* high sensitivity c-reactive protein, *Lp(a)* lipoprotein(a), *SD* standard deviation, *SES* socioeconomic status

**Fig. 1:**
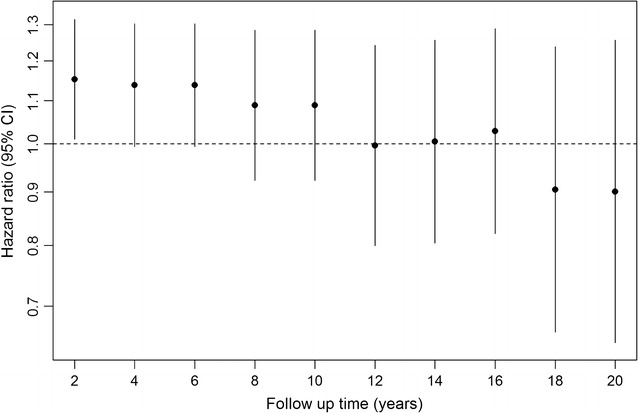
Change in the hazards ratios (95% CI) per SD lower log lipoprotein(a) for incident diabetes mellitus by follow-up time in the Bruneck Study. *CI* confidence intervals. Hazard ratios are shown for Model 3, adjusted for age, sex, alcohol consumption, BMI, smoking status, socioeconomic status, physical activity, systolic blood pressure, HDL cholesterol, log hsCRP and waist–hip ratio

**Fig. 2:**
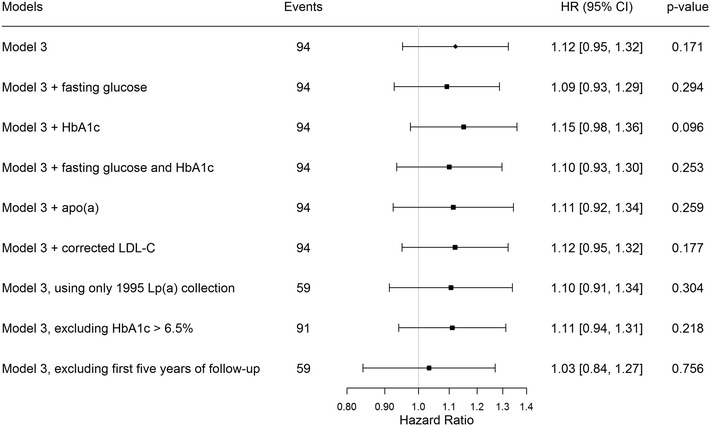
Sensitivity analyses showing the hazards ratios (HR) and 95% confidence intervals for the risk of incident type 2 diabetes per SD lower log lipoprotein(a) in the Bruneck Study. *HbA1c* hemoglobin A1c, *LDL-C* low-density lipoprotein cholesterol, *Lp(a)* lipoprotein(a). Hazard ratios are per quintile increase of standardized log lipoprotein(a). Model 3 was adjusted for age, sex, alcohol consumption, body mass index, smoking status, socioeconomic status, physical activity, systolic blood pressure, HDL cholesterol, log high sensitivity c-reactive protein and waist–hip ratio. Sensitivity analyses models as shown

The HRs per standard deviation lower log Lp(a) concentration visually decreased with longer follow-up time for Model 3 (Fig. 1), although this trend was not significant when testing the proportional-hazards assumption. We observed results similar to the principal analysis after further adjustment for markers of glycemia, apo(a) and LDL-C, when excluding participants with HbA1c >6.5%, or when accounting for possible undiagnosed prevalent cases by excluding the first 5 years of follow-up (Fig. 2). We further re-ran Model 3 using the 1995 Lp(a) measurements instead of usual levels to investigate whether the difference in how Lp(a) was measured at follow-up compared to baseline influenced the results, finding little difference compared to the main results (HR = 1.10 [0.91–1.34]).

### Meta-analysis of the association between Lp(a) quintiles and diabetes risk

A total of 1582 titles/abstracts were screened for eligibility with 47 eligible for full-text review (Additional file 1: Figure A1). Of these, five articles [5–9] reporting on four unique studies were found to be eligible for inclusion in the meta-analysis. Table 3 shows the characteristics of the included studies. All studies reported on middle-aged individuals with the median age being similar across studies and all studies used multiple data sources to verify diabetes cases (Table 3). All cohorts were sourced from the general population except for the Women’s Health Study (WHS) [5] which was comprised of health care professionals. The studies included in the meta-analysis were from Western countries and included Caucasian participants.

**Table 3:** Summary characteristics of studies included in the literature-based meta-analysis

Reference	Cohort	Country	Age, mean (SD) or median (25th–75th)	No. (%) males	N	No. incident events	Follow-up, years	Diabetes ascertainment
Bruneck study	Bruneck	Italy	Mean = 58 (11)	403 (50)	815	94	Median = 20.0	^b^
Mora et al. [5]	WHS	US	Mean = 55 (7)	0 (0)	26,746	1670	Median = 13.3	^a^
Kamstrup et al. [6]	CCHS and CGPS	Denmark	Median = 58 (47–67)	34,691 (45)	29,106	2157	Not provided	^a^
Ye et al. [9]	EPIC-Norfolk	UK	Mean = 59 (9)	8248 (45)	17,908	593	Mean = 9.8	^a^

*CCHS* Copenhagen City Heart Study, *CGPS* Copenhagen General Population Study, *EPIC-Norfolk* European Prospective Investigation of Cancer-Norfolk, *WHS* Women’s Health Study

^a^Self-report validated by linkage to other sources

^b^American Diabetes Association criteria and/or use of diabetes medication

Although the range of Lp(a) concentrations in each quintile varied between studies, all studies showed some evidence of an inverse-relationship between Lp(a) concentration and risk of type-2 diabetes, although the 95% CIs often included the null (Additional file 1: Table A1). Broadly similar results were seen in the Turkish Adult Risk Factor Study (TARF) [8] which reported risk according to tertiles, but not quintiles, of Lp(a) concentration and was not included in the meta-analysis (Additional file 1: Table A1).

**Fig. 3:**
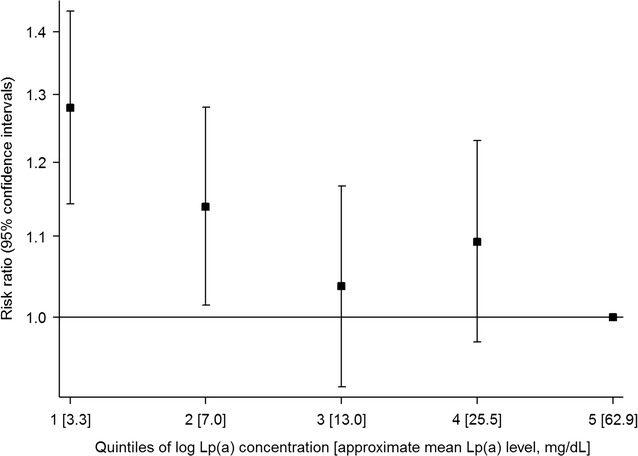
Meta-analysis of reported risk ratios for incident type-2 diabetes per quintile of Lp(a) concentration. Based on a fixed-effect meta-analysis of data from: the Bruneck study (Model 3, adjusted for age, sex, alcohol consumption, body mass index, smoking status, socioeconomic status, physical activity, systolic blood pressure, HDL cholesterol, log hsCRP and waist–hip ratio); the Copenhagen City Heart Study and the Copenhagen General Population Study (results adjusted for: age, sex, total cholesterol, HDL cholesterol, triglyceride concentrations, systolic blood pressure, body mass index, smoking status, lipid lowering therapy, and postmenopausal status and hormone replacement therapy among women); the European Prospective Investigation of Cancer-Norfolk study (results adjusted for: age, sex, body mass index, alcohol, smoking status, diastolic and systolic blood pressure, family history of diabetes, physical activity, education, total cholesterol, LDL cholesterol, prevalent cancer, CHD or stroke, antihypertension medication, lipid-lowering drugs, and CRP); and the Women’s Health Study (results adjusted for: age, race, RCT assignment, smoking status, menopausal status, postmenopausal hormone use, family history of diabetes, blood pressure, body mass index, hemoglobin A1c). Studies are weighted using the inverse variance method. Mean Lp(a) concentrations in each quintile were estimated by taking the average of the median Lp(a) concentrations in each quintile across studies, weighted by the number of participants, excluding the Women’s Health Study which did not report median Lp(a) concentrations by quintile

Figure 3 shows the risk of incident type-2 diabetes across quintiles of Lp(a) concentrations after meta-analysis. A total 74,575 participants from four prospective studies were included in the meta-analysis. The risk of incident type-2 diabetes was significantly higher in the first two quintiles (mean Lp(a) = 3.3 and 7.0 mg/dL, respectively) of Lp(a) concentration compared to quintile 5 (62.9 mg/dL). The HRs in each quintile of Lp(a) concentration, relative to quintile 5, were: 1.28 (1.14–1.43) for quintile 1; 1.14 (1.01–1.28) for quintile 2; 1.04 (0.92–1.17) for quintile 3; and 1.09 (0.97–1.23) for quintile 4. There was no evidence of heterogeneity between studies in any of the quintiles (P > 0.05 in each quintile 1–4 compared to quintile 5). The sensitivity analyses using multivariate meta-analysis with varying estimates of within-study correlations showed similar HRs in each quintile and similar overall patterns but with larger 95% confidences intervals than those observed in the main analysis (Additional file 1: Figure A2).

## Discussion

The results from this study provide evidence of an increased risk of type-2 diabetes in those with low levels of Lp(a) concentration compared to those with the highest Lp(a) concentration. Overall, there was no evidence of a linear association (P = 0.171) between Lp(a) concentration and incident type-2 diabetes in the Bruneck study. These results were robust to a series of sensitivity analyses assessing residual confounding. We also found no evidence of reverse causality after excluding the first 5 years of follow-up or when examining HRs plotted by follow-up time. Although visually the HRs appeared to increase over time, the confidence intervals overlapped between time points and the test for trend was not significant. Although there was no overall association observed in the Bruneck study, when we meta-analysed these results with results from previous prospective studies to gain statistical power, we found a higher risk of type-2 diabetes among people with Lp(a) concentrations in the lowest 2 quintiles (mean Lp(a) <7.0 mg/dL) compared to those in quintile 5 (62.9 mg/dL). This relationship was strongest in those with the lowest levels of Lp(a); those in the first quintile of Lp(a) concentration (mean Lp(a) = 3.3 mg/dL) had a 28% (HR = 1.28 [1.14–1.43]) increased risk of type-2 diabetes compared to those in the highest quintile. Overall we found a modest association between low Lp(a) levels and the risk of incident diabetes in Caucasian populations from four Western countries. The level of risk may be higher in higher-risk populations or in other ethnicities, as observed in the TARF study which found a ~twofold higher risk of incident type-2 diabetes among participants in the lowest vs. those in the highest tertile of Lp(a) concentration [8]. A large Chinese study observed an odds ratio of 1.37 for prevalent diabetes for a comparison of the lowest vs. the higher quartile of Lp(a) concentration, but was limited due to its cross-sectional design [32].

Two MR studies have previously attempted to address the question of whether Lp(a)-type-2 diabetes relationship is causal [6, 9]. Both studies showed no association between Lp(a) concentrations and risk of diabetes using the genetic variant rs10455872 as an instrumental variable. These findings were supported by a further study [33] that investigated the association between lowered Lp(a) levels and a range of cardiometabolic conditions, finding no association between a genetic risk score of 4 variants in the *LPA* gene (rs10455872, rs3798220, rs41272114 and rs143431368) and type-2 diabetes. However, a key limitation of this approach is that the main genetic variant (rs10455872) tags individuals with a broad range of Lp(a) concentrations: carriers of the variant, representing the high Lp(a) concentration group, had a median concentration of 61 mg/dL (IQR 46–82) while non-carriers had a median concentration of 11 mg/dL (IQR 5–23) [6]. The Lp(a) concentration of this non-carrier group captures the Lp(a) concentrations from quintiles 1–4. Grouping together people with such a broad range of Lp(a) concentrations may obscure associations seen only in the lowest Lp(a) concentrations and may account for the lack of association in the MR studies, as discussed by Lamina and Kronenberg [15]. In our meta-analysis, we only observed an increased risk of diabetes in people with Lp(a) concentrations in the lowest two quintiles. The rs10455872 variant cannot distinguish between these low levels of Lp(a) and is therefore not an appropriate proxy for examining the relationship between very low levels of Lp(a) concentration and diabetes risk [15].

Furthermore, Kamstrup and Nordestgaard [6] used the sum of KIV-2 repeats as a better indicator of Lp(a) plasma concentration than the rs10455872 variant, showing that individuals in the highest quintile of the genetically determined sum of KIV-2 repeats (that are more likely to have low or medium Lp(a) concentrations), have a 16% increased risk of diabetes compared to quintiles 1–4 of KIV-2 repeats (OR 1.16 [1.05–1.28]). The genetic instrument of the sum of KIV-2 repeats is not a perfect measure since it is a sum of two alleles which can be quite heterogeneous (one allele with a low and one with a high number of KIV-2 repeats, or both with a medium sized number of repeats) and it ignores that 30–50% of individuals express only one apo(a) isoform in plasma although they are clearly heterozygote at the DNA level. In the present study we found no evidence of an association between isoform size and diabetes risk (HR = 1.01 [0.97–1.06] for large isoform size compared to small isoforms), possibly due to a lack of power. A difference in the methods is that we used gel electrophoresis in the Bruneck Study that gives allele-specific information, whereas the PCR method used in the Copenhagen studies [6] summates the size of both alleles and likely weakens the power of such analyses due to regression to the mean of apo(a) isoform size. Furthermore, this method has been shown to correlate only modestly with allele specific methods of apo(a) isoform determination [34].

The causality of the relationship between Lp(a) concentration and diabetes risk has not yet been sufficiently investigated and further studies using genetic variants that selectively tag low concentrations of Lp(a) are needed. Furthermore, the mechanisms through which Lp(a) might be inversely associated with diabetes risk are not yet clear. There is some evidence that Lp(a) is a marker of, or might be involved in, the development of insulin resistance. Previous studies have shown that lower levels of Lp(a) concentration and large apo(a) isoform size are associated with higher insulin levels [35], and that insulin can inhibit the transcription of apo(a) in monkey hepatocytes [36]. It has also been speculated [37] that the inverse association of Lp(a) with diabetes may result from increased oxidative stress, oxidation of Lp(a) phospholipids, and subsequent low-grade inflammation and autoimmune activation. Recent evidence demonstrated causal inverse relationships for LDL cholesterol [38] and inverse associations of LDL particle size [39] with incident type-2 diabetes, and it may be hypothesised that the association of Lp(a) is partly mediated via the LDL pathway.

With the recent development of drugs that selectively lower Lp(a) levels [10], Lp(a) could become a clinical target for reducing CVD risk. To date, results from three trials have shown no effect on glucose with significant lowering of Lp(a) to >90% [10, 40]. Although the effect of such treatment on diabetes risk is not known, Lp(a) lowering therapy would likely only be used in people with highly elevated Lp(a) levels and few patients would be treated to low Lp(a) levels of <7 mg/dL where the risk seems to increase in the meta-analysis results. This is not the first example of a contrasting relationship between a lipid subclass and risk of coronary heart disease (CHD) and diabetes. Evidence from MR studies [41] and studies of familial hypercholesterolemia (a genetic condition resulting in high levels of plasma LDL-C) [42] show a causal relationship between LDL-C and increased CHD risk but a decreased risk of type-2 diabetes. While statins, which lower LDL-C levels, have been shown to substantially decrease the risk of CVD, there is evidence that they increase the risk of type-2 diabetes [43]. However, this increased absolute risk of diabetes is considered to be low and does not outweigh the benefits of statins in reducing CVD events [43]. Further evidence of this is provided by a study of familial hypobetalipoproteinemia which showed no increase in type-2 diabetes among people with genetically determined low levels of LDL-C [42]. A recent MR study using genetic variants for proprotein convertase subtilisin-kexin type 9 (PCSK9) and 3-hydroxy-3-methylglutaryl-coenzyme A reductase (HMGCR), that mimic the effects of lowering LDL-C levels, showed an increased risk of diabetes with lower LDL-C. However, both prevalent and incident diabetes cases were analysed together, and, notably, among people with normal fasting glucose levels at baseline, there was no increased risk of incident diabetes with LDL-C lowering [44].

The keys strengths of our study using the Bruneck data include: long follow-up time (20 years), representativeness of the general community and availability of a wide range of measured clinical factors, allowing us to accurately ascertain type-2 diabetes as well as investigate the role of reverse-causality and residual bias. Although the Bruneck sample size was small and the precision of the estimated association measures was low, we were able to meta-analyse these results with previous prospective studies to provide a more precise estimate of the association between low levels of Lp(a) concentration and risk of type-2 diabetes. Lp(a) concentration is largely stable over time and, to account for any residual regression dilution bias, we estimated long-term usual levels of Lp(a). We were unable to account for any effect of fasting status on Lp(a) concentrations since this varied across the included studies and was not always reported. However, it has been previously found that normal food intake does not affect median Lp(a) levels [45], indicating that fasting status is unlikely to have impacted the effect sizes estimated in the meta-analysis. No information on within-study variability was available for the previously published prospective studies which limited our ability to account for correlations within the Lp(a) quintiles in the meta-analysis. However, the measures of association estimated in the sensitivity analyses using multivariate meta-analysis were consistent with the main results. Weighted mean Lp(a) concentration in each quintile for the meta-analyses could only be estimated using data from 3/4 studies as median Lp(a) levels per quintile were not reported for one of the studies (WHS). We were limited to using available summary data on the Lp(a)-diabetes relationship reported in quintiles in previous studies, and were unable to undertake more sophisticated analyses to assess the shape of the relationship. Furthermore, there were inconsistencies between studies in which confounders were adjusted for, however these adjustments slightly strengthened, rather than weakened, the effect estimates in most studies.

## Conclusions

Our study provides evidence to support the hypothesis that Lp(a) concentration is inversely associated with development of type-2 diabetes, in people without previous diabetes. That we only observed an increased risk of diabetes in people with Lp(a) concentrations in the lowest two quintiles (~mean Lp(a) levels of <7 mg/dL) suggest that the use of Lp(a) lowering therapies would not be in conflict with these findings if provided therapies do not lower Lp(a) levels beyond those observed in these lowest two quintiles.

## Additional file

**Additional file 1.** Online appendix.
