# Supplementary material for: Lipoprotein(a) and incident type-2 diabetes: results from the prospective Bruneck study and a meta-analysis of published literature
Source: Cardiovasc Diabetol. 2017 Mar 21;16:38. doi: 10.1186/s12933-017-0520-z (PMC5359972; doi:10.1186/s12933-017-0520-z)
Supplement: Supplementary file 1 — Additional file 1. Online appendix. [file 12933_2017_520_MOESM1_ESM.pdf]

Lipoprotein(a) and Incident Type-2 Diabetes: Results from the Prospective Bruneck Study and a Meta-analysis of Published Literature

Online appendix

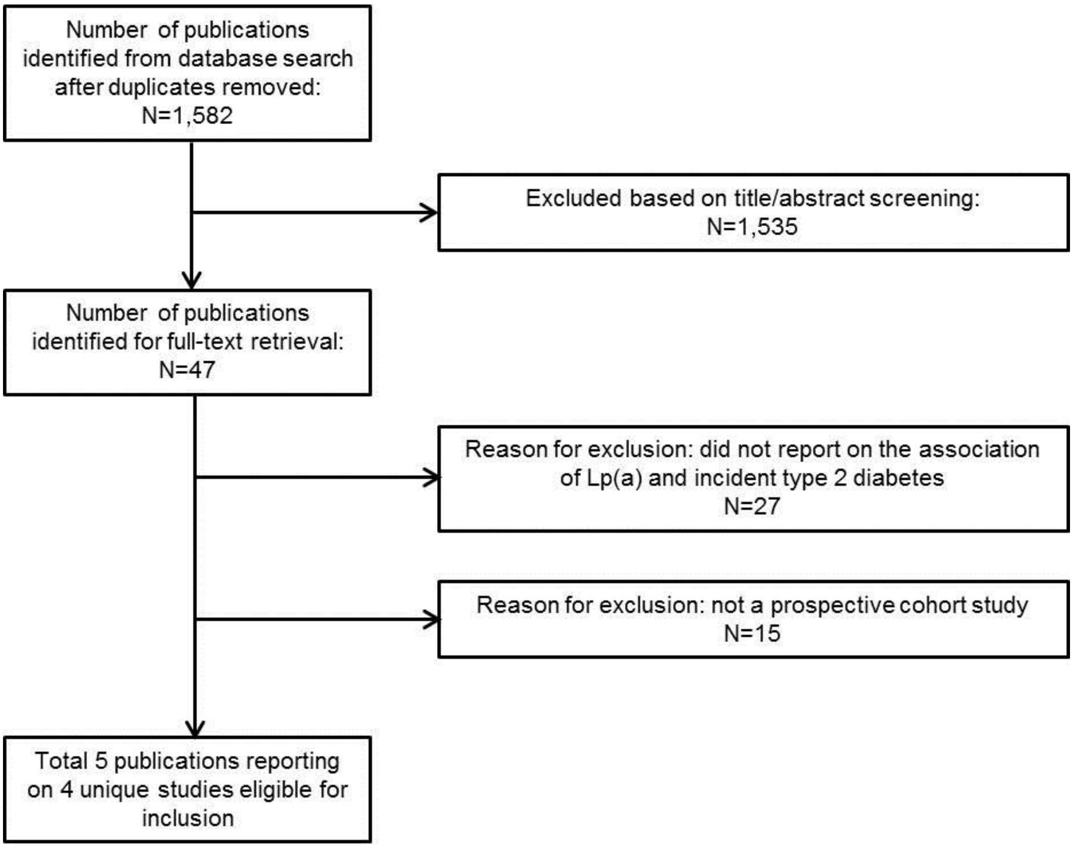

Figure A1: Flow-chart showing study selection for the literature-based meta-analysis

A) Within-study correlation estimated at 0.2

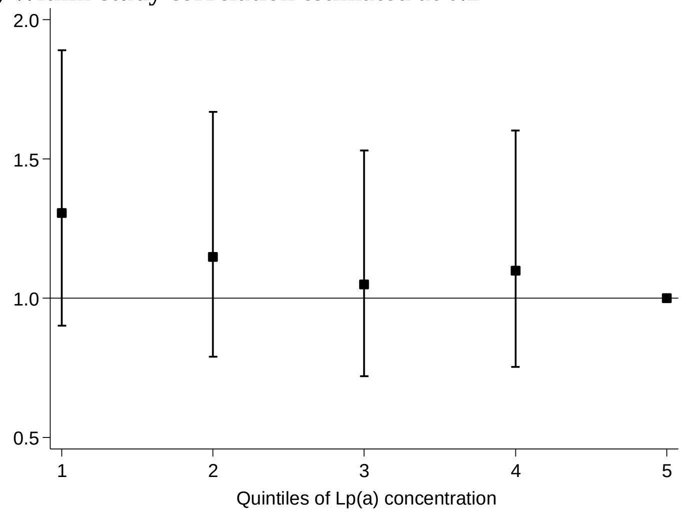

B) Within-study correlation estimated at 0.5

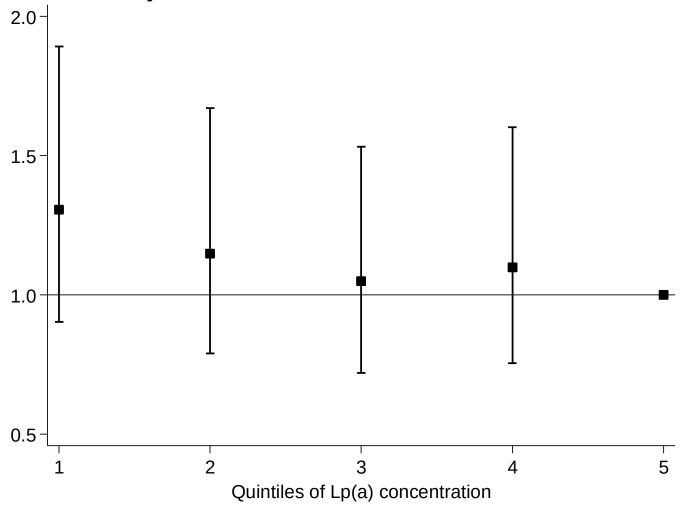

C) Within-study correlation estimated at 0.8

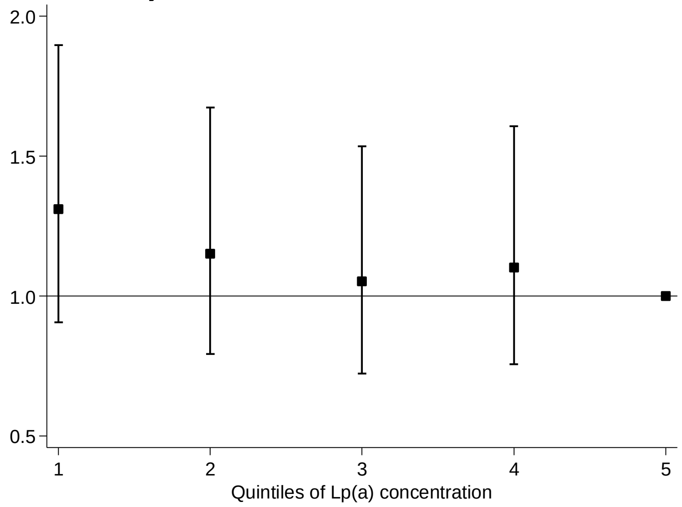

Figure A2: Sensitivity analysis showing the shape plots of the risk of type-2 diabetes by Lp(a) quintiles Results are from three multivariate meta-analyses with varying levels of estimated within-study correlation: A) 0.2; B) 0.5; and C) 0.8.

Based on multivariate meta-analyses of data from: the Bruneck study (Model 3); the Copenhagen City Heart Study; the Copenhagen General Population Study; the European Prospective Investigation of Cancer – Norfolk study; and the Women's Health Study.

Table A1: Effect sizes and 95% confidence intervals extracted from published studies

	WHS study		CCHS and CGPS studies		EPIC-Norfolk study	
	**HR (95% CI)**	**Median (range) Lp(a) conc.***	**OR (95% CI)**	**Median (range) Lp(a) conc.***	**HR (95% CI)**	**Median (range) Lp(a) conc.***
**Quintiles**						
1	Reference	(<3.9)	1.26 (1.09, 1.45)	3 (1-4)	Reference	3.9 (2.0-5.3)
2	0.87 (0.75, 1.01)	(3.9-8.4)	1.17 (1.01, 1.36)	7 (5-11)	0.66 (0.50, 0.86)	7.1 (5.3-9.0)
3	0.80 (0.68, 0.93)	(8.5-16.6)	1.04 (0.90, 1.21)	14 (10-19)	0.70 (0.54, 0.92)	11.6 (9.0-15.4)
4	0.88 (0.76, 1.02)	(16.7-45.3)	1.05 (0.90, 1.22)	28 (20-35)	0.75 (0.58, 0.97)	21.8 (15.4-35.4)
5	0.78 (0.67, 0.91)	(>45.3)	Reference	69 (55-99)	0.64 (0.49, 0.85)	53.5 (35.4-175.0)

CCHS= Copenhagen City Heart Study; CGPS= Copenhagen General Population Study; EPIC-Norfolk= European Prospective Investigation of Cancer – Norfolk; WHS=Women's Health Study. *Lp(a) concentration given as mg/dL.
